# The *Caenorhabditis elegans* Oxidative Stress Response Requires the NHR-49 Transcription Factor

**DOI:** 10.1534/g3.118.200727

**Published:** 2018-10-08

**Authors:** Queenie Hu, Dayana R. D’Amora, Lesley T. MacNeil, Albertha J. M. Walhout, Terrance J. Kubiseski

**Affiliations:** * Department of Biology; ‡ Program of Neuroscience, York University, Toronto, Ontario, Canada; † Program in System Biology and Program in Molecular Medicine, University of Massachusetts Medical School, Worcester, MA

**Keywords:** *C. elegans*, NHR-49, oxidative stress, reactive oxygen species, SKN-1

## Abstract

The overproduction of reactive oxygen species (ROS) in cells can lead to the development of diseases associated with aging. We have previously shown that *C. elegans* BRAP-2 (Brca1 associated binding protein 2) regulates phase II detoxification genes such as *gst-4*, by increasing SKN-1 activity. Previously, a transcription factor (TF) RNAi screen was conducted to identify potential activators that are required to induce *gst-4* expression in *brap-2(ok1492)* mutants. The lipid metabolism regulator NHR-49/HNF4 was among 18 TFs identified. Here, we show that knockdown of *nhr-49* suppresses the activation of *gst-4* caused by *brap-2* inactivation and that gain-of-function alleles of *nhr-49* promote *gst-4* expression. We also demonstrate that *nhr-49* and its cofactor *mdt-15* are required to express phase II detoxification enzymes upon exposure to chemicals that induce oxidative stress. Furthermore, we show that NHR-49 and MDT-15 enhance expression of *skn-1a/c*. These findings identify a novel role for NHR-49 in ROS detoxification by regulating expression of SKN-1C and phase II detoxification genes.

In nature, cells may encounter both exogenous and endogenous stressors that can alter normal physiological processes. One such form of stress, oxidative stress, is caused by reactive oxygen species (ROS) that have an ability to threaten cell survival. The imbalance between ROS and protective detoxification enzymes can lead to extensive oxidative damage to macromolecules such as DNA, lipids, and proteins (Buonocore *et al.* 2010; Finkel 2011). To protect cells against oxidative stress, organisms have developed lines of defense to cope with changes in levels of ROS to maintain homeostasis. The regulation of detoxification genes frequently involves complex transcriptional regulatory networks and, as a result, increases the potential for cross talk between stress signaling pathways. The induction of detoxification genes could be the result of cooperation between multiple transcription factors (TFs) (Rahman 2007). Therefore, in order to understand the genetic regulatory network involved in maintaining cellular integrity, it is vital to identify the factors that regulate stress response genes and promote survival.

Like mammals, the nematode *C. elegans* has well-defined stress defense systems for protection from toxic compounds (Van Raamsdonk and Hekimi 2010). These signaling pathways, and their modes of regulation, share evolutionary conservation with their mammalian counterparts (Tissenbaum 2015). Thus, *C. elegans* offers a suitable model to dissect the gene regulatory network involved in the expression of stress response genes. In recent years, increased attention has been given to the conserved TFs DAF-16/FOXO and SKN-1/Nrf2 due to their associated roles in response to oxidative stress and lifespan extension in *C. elegans* (Kenyon *et al.* 1993; Murphy *et al.* 2003; An and Blackwell 2003; Blackwell *et al.* 2015). These factors regulate the transcription of essential detoxification genes such as *sod-3* and *gst-4* to promote resistance to oxidative stress (Oliveira *et al.* 2009; Wang *et al.* 2010; Shore and Ruvkun 2013). Although the signaling pathways and mechanisms that control the nuclear localization of both TFs have been revealed, the oxidative stressors that activate these pathways remain poorly understood.

Mammalian Brap2 (Brca1 associated binding protein 2 or Brap as listed in the HUGO database) was first identified as a Brca1 binding protein that occludes the Brca1 nuclear localization motif, preventing it from translocating to the nucleus, and subsequently been shown to act as a cytoplasmic retention protein for a number of proteins (Li *et al.* 1998; Asada *et al.* 2004; Chen *et al.* 2009; Davies *et al.* 2013). Brap2 is also a Ras-responsive E3 ubiquitin ligase that functions as a modulator of the Ras signaling pathway by facilitating activation of Erk upon cell stimulation (Ory and Morrison 2004; Matheny and White 2006, 2009). Work in *C. elegans* has shown that loss of functional *brap-2* causes hypersensitivity to hydrogen peroxide or paraquat (Koon and Kubiseski 2010). Furthermore, we performed an RNAi screen and found that BRAP-2 regulates the TFs SKN-1 and ELT-3 for the induction of phase II detoxification genes (Hu *et al.* 2017). The study also revealed that NHR-49, a TF with a role in regulating expression of proteins involved in lipid synthesis, suppresses *gst-4* expression in *brap-2(ok1492)*.

Nuclear hormone receptors (NHRs) are TFs that are generally activated by lipophilic hormones (Antebi 2015). NHR-49 is a key regulator of the “fasting response” that leads to changes in fatty acid metabolism for both basal or starvation states (Van Gilst *et al.* 2005a; b; Ratnappan *et al.* 2014). Loss of *nhr-49* causes an increase in body fat and stimulates an impaired nutritional response. Additionally, *nhr-49(lf)* mutants exhibit a shortened lifespan caused by an imbalance in lipid composition that leads to lipotoxicity (Pathare *et al.* 2012; Grants *et al.* 2015). NHR-49 functions together with the mediator MDT-15 and removal of *mdt-15* fails to stimulate *nhr-49*-dependent fasting response genes (Taubert *et al.* 2006). MDT-15 also interacts with SKN-1 to facilitate oxidative metabolism and promote lifespan in an *nhr-49* independent manner (Goh *et al.* 2014; Pang *et al.* 2014). NHR-49 is a homolog of hepatocyte nuclear factor 4 alpha (HNF4α), yet appears to function in a manner similar to that of the related proliferator-activated receptor alpha (PPARα) in regulating fatty acid uptake, lipoprotein transport, and mitochondrial and peroxisomal β-oxidation (Contreras *et al.* 2013; Ratnappan *et al.* 2014).

Here we report that NHR-49 is essential for the SKN-1 dependent expression of phase II detoxification genes. The elucidation of SKN-1 co-activators, such as NHR-49 and MDT-15, for the control of this transcriptional regulation provides further insight in the complex gene regulatory network that controls stress gene expression.

## Materials and Methods

### C. elegans Strains

All *C. elegans* strains were maintained as described by Brenner (Brenner 1974). Double mutant strains were generated according to standard protocols. Unless stated otherwise, worm strains were provided by the *Caenorhabditis* Genetics Center (CGC, University of Minnesota) and the National Bioresource Project (Tokyo, Japan). Strains used in this study were as follows: *Bristol strain* N2, *dvIs19* (CL2166), *brap-2(ok1492)* (YF15), *nhr-49(ok2165)* (YF127), *brap-2(ok1492);nhr-49(ok2165)* (YF126), *mdt-15(tm2182)* (XA7702), *brap-2(ok1492);mdt-15(tm2182)* (YF131), *nhr-49(nr2041)* (STE68), *nhr-49(et7)* (QC120), *nhr-49(et8)* (QC121), *nhr-49(et13)* (QC126) and *wdr-23(tm1817)* (YF208).

### RNAi Treatment

RNAi was performed as described previously (MacNeil *et al.* 2015; Hu *et al.* 2017). Bacteria expressing dsRNA was grown on nematode growth medium (NGM) containing 0.4 mM IPTG, 100 µg/mL ampicillin, and 12.5 µg/mL tetracycline. Synchronized worms were grown on RNAi plates. Animals were collected at the L4 stage and analyzed for GFP expression by confocal microscope or used for RNA isolation.

### Fluorescence microscopy

Live L4 *gst-4p*::*gfp* expressing worms were anesthetized using 2 mM Levamisole (Sigma L9756) and mounted on 2% agarose pad. Images of fluorescent worms were taken using a Zeiss LSM 700 confocal laser-scanning microscope with Zen 2010 Software.

### Paraquat/Arsenite/Acrylamide Treatment

For each strain, synchronized worms were grown on NGM plates and were collected in M9 buffer at the L4 stage. Sodium arsenite (Sigma #35000) or paraquat (Sigma #856177) was diluted in M9 buffer to a final concentration of 5 mM and 100 mM, respectively. Collected worms were treated with each drug at room temperature for 2 hr followed by RNA isolation and qPCR. Each experiment was completed in triplicate. For acrylamide treatment, three independent lines of N2 or *nhr-49(ok2165)* synchronized L1-stage animals were grown on acrylamide (500 mg/L) or control plates (without acrylamide) for 48 hr at 20° as described (Hasegawa *et al.* 2008). L4 animals were then collected using M9 buffer and stored at -80° until RNA isolation.

### Quantitative PCR

Quantitative PCR was used to measure mRNA levels as described previously (Hu *et al.* 2017). qPCR data were derived from 3 independent replicates and were analyzed using the comparative method (ΔΔCt). Results were graphed and the relative expression of each strain was compared to N2. The endogenous control used for normalization was *act-1*. Primer sequences were previously described (Hu *et al.* 2017).

### Statistics

Statistical significance was determined using unpaired student’s *t*-test when two means were compared and corrected for multiple comparisons using the Holm-Sidak method. P values of <0.05 were taken to indicate statistical significance. Error bars represent +/− standard error of the mean.

### Data availability

Strains are available upon request or through the *Caenorhabditis* Genetics Center (CGC). All the data necessary for confirming the conclusions presented in the article are represented fully within the article.

## Results

**Figure 1:**
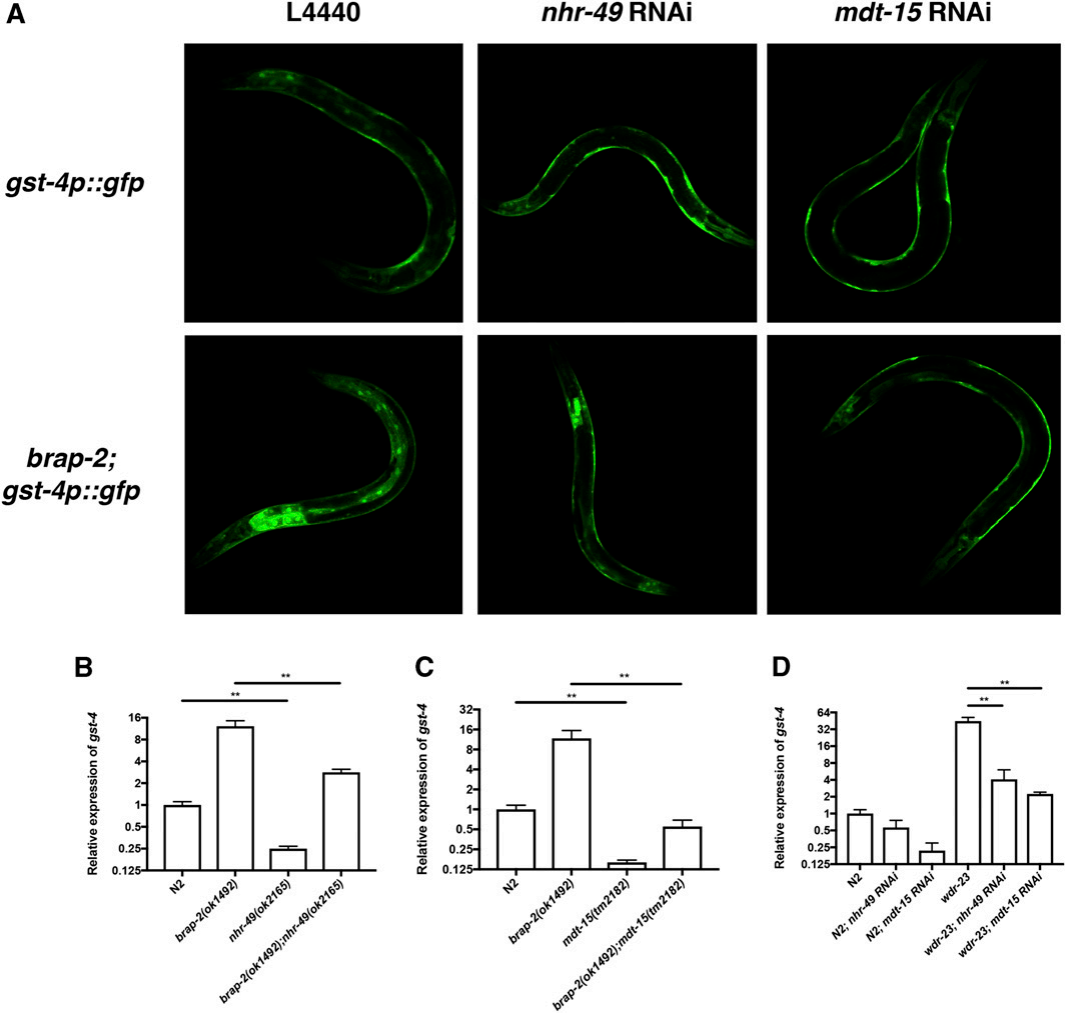
*nhr-49* and *mdt-15* are essential for enhanced *gst-4* expression. *(A) gst-4p*::*gfp* and *brap-2(ok1492);gst-4p*::*gfp* worms were treated with *nhr-49* or *mdt-15* RNAi followed by examination of GFP expression using confocal microscopy. Representative GFP images show *brap-2(ok1492);gst-4p*::*gfp* worms grown in either *nhr-49* or *mdt-15* RNAi causes a reduction of *gst-4p*::*gfp* expression. Twenty worms were examined and one representative worm shown. *(B*, *C)* Following RNA extraction, *gst-4* mRNA levels were measured by qPCR. *gst-4* expression is reduced in both *(B) brap-2(ok1492);nhr-49(ok2165)* and *(C) brap-2(ok1492);mdt-15(tm2181)*. *(D) wdr-23(tm1817)* worms grown in either *nhr-49* or *mdt-15* RNAi showed a significant decrease in *gst-4* mRNA levels as measured by qPCR. *P* < 0.05*, *P* < 0.01**, *P* < 0.001***.

The TF NHR-49 and its mediator subunit MDT-15 are required for phase II detoxification gene expression in *brap-2(ok1492)* mutants. Previously, we showed that C. elegans BRAP-2 is required to regulate the TF SKN-1 to induce phase II detoxification gene expression. To further the study of this regulatory network, a TF specific RNAi screen was conducted to identify regulators of *gst-4* expression in *brap-2*(ok1492) mutants (Hu *et al.* 2017). Our screen identified 18 TFs that decreased GFP expression in *brap-2(ok1492)* mutants, including *nhr-49*. To validate this result, L4 *brap-2(ok1492)* mutants carrying the gst-4p::gfp transgene were fed *nhr-49* RNAi and GFP expression was examined. The *nhr-49* RNAi treated *brap-2(ok1492);gst-4p::gfp* animals displayed lower GFP expression compared to the RNAi vector control (Figure 1A). We also examined gst-4 expression by qPCR in *brap-2(ok1492);nhr-49(ok2165)* double mutant and found an ∼75% reduction of *gst-4* mRNA (Figure 1B). Taken together, this indicates that *nhr-49* is required for *gst-4* expression.

NHR-49 requires the mediator MDT-15 to modulate target gene expression and lipid composition (Taubert *et al.* 2006). MDT-15 contributes to detoxification gene induction and, together with its interacting partner SKN-1, is required for the oxidative stress response (Taubert *et al.* 2008; Goh *et al.* 2014; Grants *et al.* 2015). To determine if MDT-15 is also needed to up-regulate *gst-4* expression in *brap-2(ok1492)*, we knocked down *mdt-15* in *brap-2(ok1492);gst-4p*::*gfp* animals and monitored effects on GFP expression. Additionally, a *brap-2(ok1492);mdt-15(tm2182)* double mutant was generated and *gst-4* mRNA levels were measured using qPCR. As expected, loss of *mdt-15* resulted in a reduction in *gst-4* levels (Figure 1A and 1C), indicating that MDT-15 also plays a role in *gst-4* regulation in the *brap-2(ok1492)* mutant, consistent with previous studies showing that MDT-15 is required for oxidative stress response. The *C. elegans* protein WDR-23 functions to prevent the accumulation of SKN-1 in the nucleus by targeting it for degradation (Choe *et al.* 2009). Since *gst-4* expression is increased in *wdr-23* mutants, and *mdt-15* is required for this increase (Goh *et al.* 2014; Wu *et al.* 2016), we wanted to determine if *nhr-49* was also required in this context. We knocked down *nhr-49* using RNAi in the *wdr-23(tm1817)* mutant and again found a significant reduction in *gst-4* mRNA levels (Figure 1D).

**Figure 2:**
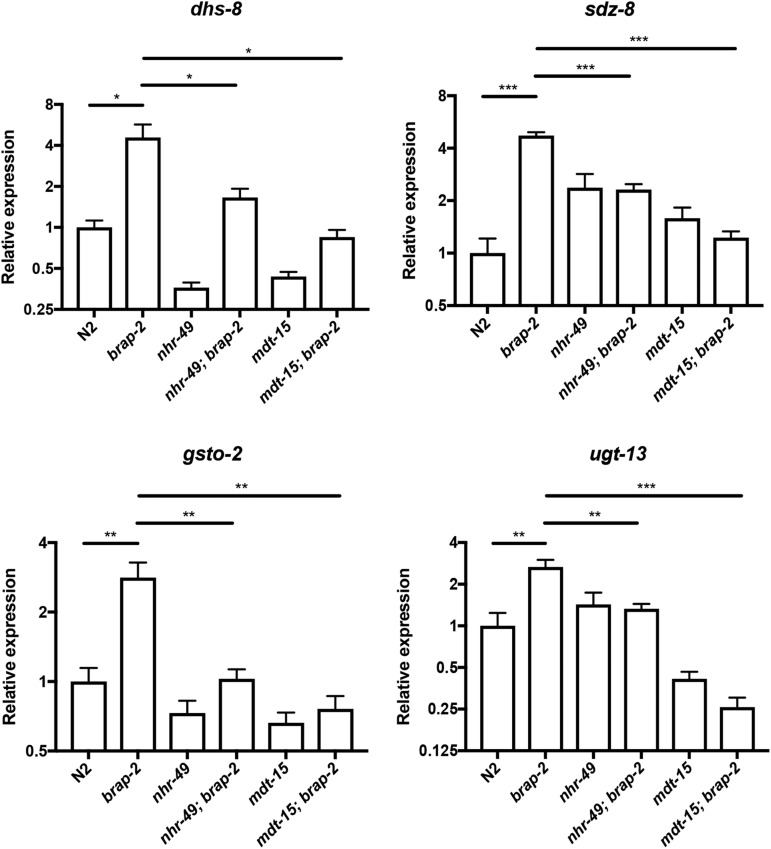
The mRNA levels of four phase II detoxification genes (log2 scale) were compared between wild type and *brap-2(ok1492)* mutants in the presence and absence of *nhr-49(ok2165)* or *mdt-15(tm2182)* alleles. A significant decrease in mRNA levels was found in all four phase II genes tested (*dhs-8*, *sdz-8*, *gsto-2*, and *ugt-13*) upon *nhr-49* or *mdt-15* mutation in *brap-2(ok1492)*. *P* < 0.05*, *P* < 0.01**, *P* < 0.001***.

To confirm the requirement of *nhr-49* and *mdt-15* in phase II detoxification in *brap-2(ok1492)*, we examined four additional phase II genes (*dhs-8*, *sdz-8*, *gsto-2* and *ugt-13*). The expression of all four genes was significantly decreased in *brap-2(ok1492)* when either *nhr-49* or *mdt-15* was absent (Figure 2). Therefore, NHR-49 and MDT-15 are required for activation of phase II detoxification genes in *brap-2(ok1492)* animals.

**Figure 3:**
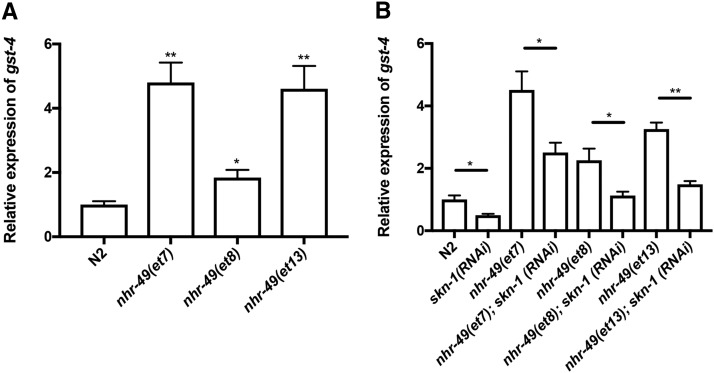
*skn-1* is required to regulate *gst-4* expression in gain-of-function *nhr-49* worms. RNA was extracted from synchronized L4 worms followed by quantification of *gst-4* transcript levels using qPCR. *(A)* Three gain-of-function *nhr-49* strains were used to examine *gst-4* mRNA expression. Results display an increase in *gst-4* mRNA by at least twofold. *(B)* The *nhr-49* gain-of-function strains were treated with control (L4440) or *skn-1* RNAi and *gst-4* mRNA levels were quantified. A reduction of *gst-4* was seen with *skn-1* knockdown compared to strains fed on the L4440 control; *P* < 0.01**, *P* < 0.05* *vs.* N2 in *(A)*; *P* < 0.001***, *P* < 0.01** *vs.* L4440 control in *(B)*.

Since *nhr-49* is required for the expression of the phase II detoxification gene *gst-4*, we hypothesized that an increase in *gst-4* transcript levels would be seen with gain-of-function (gof) alleles of *nhr-49* (Lee *et al.* 2016). Indeed, we observed a 1.8 to 4.8-fold increase in *gst-4* expression in *nhr-49(gof)* mutants (Figure 3A). We next asked if this increase in *gst-4* expression required SKN-1. *skn-1* was knocked down in *nhr-49(gof)* strains and *gst-4* mRNA levels were measured using qPCR. The depletion of *skn-1* caused a decrease in *gst-4* expression when compared to the untreated RNAi control (Figure 3B). Taken together, these observations are consistent with NHR-49 and SKN-1 being required to promote the induction of phase II detoxification genes.

**Figure 4:**
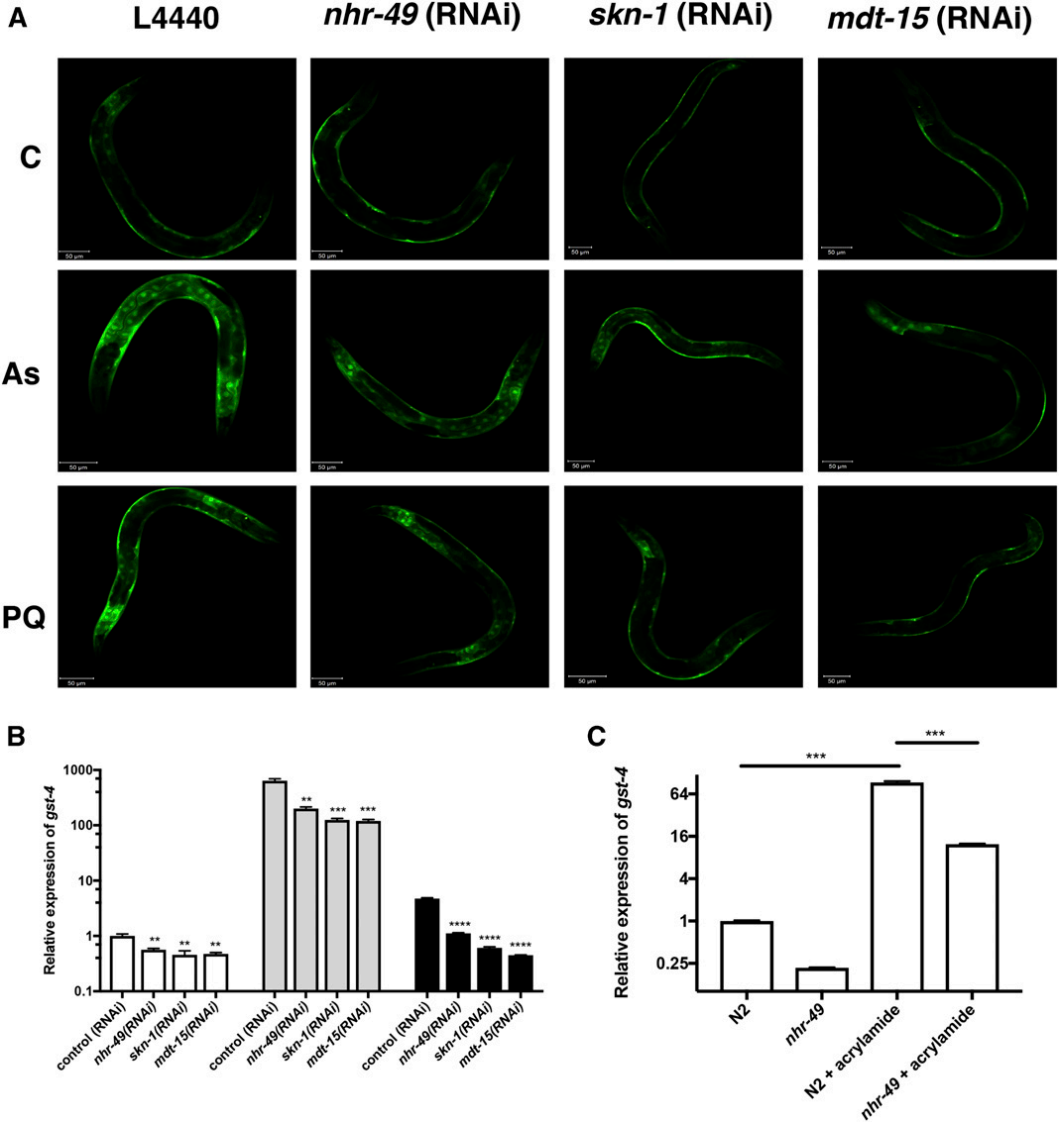
*skn-1*, *mdt-15*, and *nhr-49* are essential to induce the expression of the arsenite, paraquat, and acrylamide responsive gene *gst-4* in L4 worms. *(A)* Synchronized *gst-4p*::*gfp* worms were grown in the control (L4440), *skn-1* RNAi, *nhr-49* RNAi or *mdt-15* RNAi followed by exposure to M9 buffer (C), 5 mM sodium arsenite (As) or 100 mM paraquat (PQ) for 2 hr at L4 stage. Worms were recovered on NGM plates for 1 hr and the GFP expression was examined using confocal microscopy. Results show a reduction in GFP levels in RNAi treated worms. Twenty worms were examined and figures depict one worm. *(B)* Synchronized L4 stage worms were collected after RNAi exposure and drug treatment, followed by RNA extraction. *gst-4* mRNA transcript levels were quantified using qPCR. Values are relative to the control (RNAi) and normalized to the endogenous control *act-1*. The knock down of either *nhr-49*, *skn-1* or *mdt-15* exhibit a decrease in *gst-4* mRNA expression after mock treatment (white bars), arsenite (gray bars) or paraquat (black bars) treatment in comparison to the N2 drug-treated control. *(C)* Synchronized wild type and *nhr-49(ok2165)* animals were grown for 48 hr at 20°C on seeded plates with or without acrylamide and harvested for RNA extraction. *gst-4* mRNA transcript levels were quantified using qPCR. *P* < 0.001*** *P* < 0.01** *vs.* untreated and treated controls in *(B)*, *P* < 0.001*** *vs.* wild type in *(C)*.

In *C. elegans*, oxidative stress can be induced using sodium arsenite or paraquat, both of which significantly increase the expression of phase II detoxification enzymes in wild type worms (Oliveira *et al.* 2009). Previously, it was shown that *skn-1* and *mdt-15* are required to upregulate phase II detoxification genes upon arsenite induction (Goh *et al.* 2014). Therefore, we asked if NHR-49 activates *gst-4* in response to oxidative stress. We grew synchronized wild type worms containing *gst-4p*::*gfp*, knocked down *nhr-49*, *mdt-15* or *skn-1*, and exposed the animals to 5 mM sodium arsenite or 100 mM paraquat for two hours after which GFP expression was examined using confocal microscopy. In wild type animals, *gst-4p*::*gfp* expression was increased upon exposure to arsenite or paraquat and this increase was reduced upon *nhr-49*, *mdt-15* or *skn-1* RNAi (Figure 4A). qPCR was performed to quantify levels of *gst-4* and a reduction in mRNA was observed in *nhr-49*, *skn-1,* and *mdt-15* RNAi treated animals (Figure 4B). We also examined *gst-4* expression following acrylamide exposure over 48 hr and found a significant decrease in *gst-4* mRNA levels in the *nhr-49(ok2165)* strain relative to wild type (Figure 4C). These results demonstrate an important role for *nhr-49*, *mdt-15*, and *skn-1* in the regulation of ROS detoxification genes upon exposure to oxidative stress.

**Figure 5:**
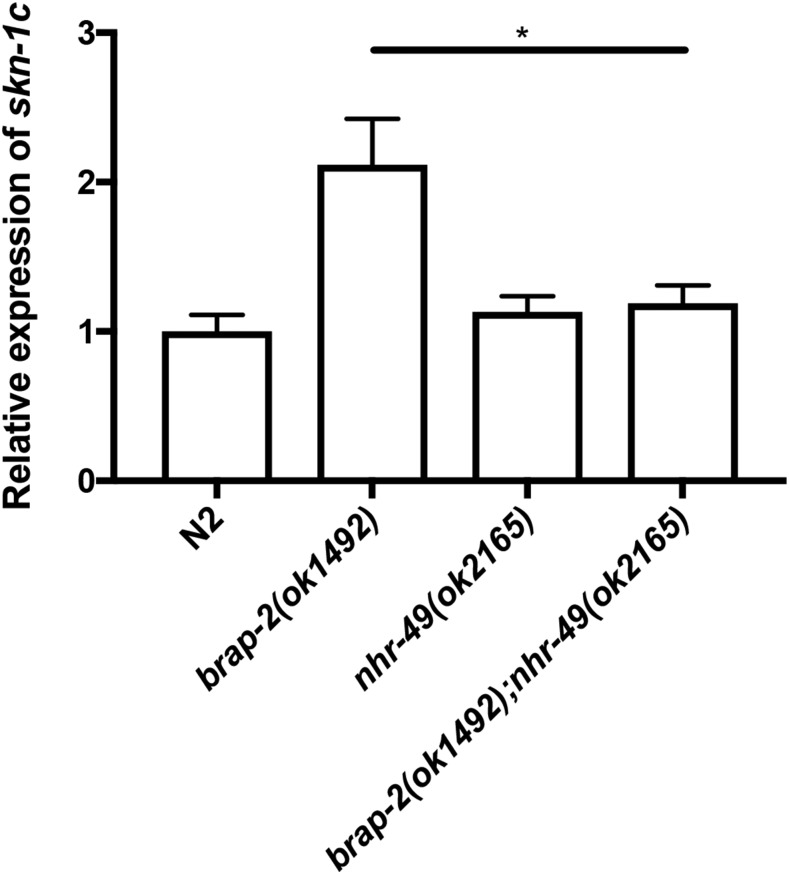
Functional NHR-49 and MDT-15 are required to promote *skn-1* transcriptional activity. *(A)* Relative *skn-1* mRNA expression was quantified in *nhr-49(ok2165)* and *brap-2(ok1492);nhr-49(ok2165)* mutant strains using qPCR. The worms displayed a reduction of *skn-1c* in the double mutant to wild type levels. *P* < 0.001***.

*NHR-49 is required to activate skn-1 transcription*. Previously, we found that *brap-2(ok1492)* mutants have higher levels of *skn-1* mRNA than wild type animals (Hu *et al.* 2017). We asked if NHR-49 is required for the increased *skn-1* expression observed in *brap-2* mutants. We measured *skn-1* mRNA levels in *brap-2;nhr-49* deletion strains and observed a twofold increase of *skn-1*, and the loss of *nhr-49* in *brap-2(ok1492)* decreases the amount of *skn-1* mRNA, restoring it to wild type levels (Figure 5). This indicates that *nhr-49* plays a role in inducing *skn-1* expression in the *brap-2(ok1492)* strain under oxidative stress conditions yet is not required for its basal expression.

## Discussion

In this study we further investigated the role of BRAP-2 and SKN-1 in the *C. elegans* oxidative stress response. We show that NHR-49 (the *C. elegans* PPARα/HNF4 homolog) and the mediator MDT-15 (MED15 homolog) are essential to regulate the SKN-1 dependent stress response in *brap-2(ok1492)*. We have found that NHR-49, MDT-15, and SKN-1 co-regulate the induction of *gst-4* and *skn-1*. By investigating mRNA expression of designated target genes in null mutants, we were able to provide evidence that NHR-49 not only participates in fat metabolism but is also a key player in the oxidative stress response. Interestingly, neither null mutations of *nhr-49* nor *mdt-15* result in the complete loss of *gst-4* (or phase II detoxification gene) expression in the *brap-2(ok1492)* strain, indicating that additional regulators exist.

**Figure 6:**
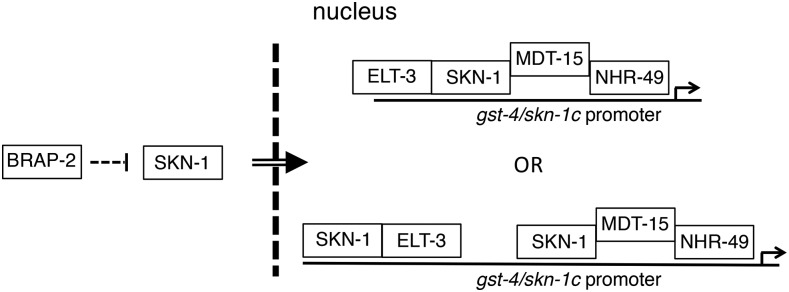
Proposed model of NHR-49 in the regulation of phase II detoxification gene *gst-4*. The induction of oxidative stress in *brap-2(ok1492)* mutant worms activates PMK, which then phosphorylates SKN-1 for nuclear translocation. SKN-1 then binds to NHR-49/MDT-15 in the nucleus to induce *gst-4* expression. This complex can also create a feed-forward loop through *skn-1c* promoter binding to up regulate its own transcription, thereby enhancing both the SKN-1C response to oxidative stress and its target genes.

Although we do not show a direct interaction of these regulators with the *gst-4* or *skn-1* promoters, it has been reported that MDT-15 can interact with both SKN-1 and NHR-49 independently (Goh *et al.* 2014). This suggests that it is possible that MDT-15 acts as a bridge between SKN-1 and NHR-49 (Figure 6). Previously, we showed that SKN-1 and the GATA factor ELT-3 heterodimerize and promote expression of phase II detoxification genes (Hu *et al.* 2017). It will be of interest to determine whether MDT-15 interacting with SKN-1 is independent of ELT-3, and if a transcriptional complex consisting of ELT-3, SKN-1, MDT-15, and NHR-49 forms to induce *gst-4* in *brap-2* mutant animals.

The focus of NHR-49 research in *C. elegans* has been to explore its role in regulating fatty acid metabolism. In addition, a study suggested NHR-49 also helps to promote lifespan in animals lacking a germline by controlling lipid metabolic pathways (Ratnappan *et al.* 2014). *nhr-49* mutants are hypersensitive to various stress inducing molecules including arsenite and tert-butyl hydroperoxide (Goh *et al.* 2014; Pang *et al.* 2014). NHR-49 promotes fatty acid β-oxidation, increases acetyl CoA levels and enhances activity of the electron transport chain, effects that are expected to increase ROS levels. Here we show that NHR-49 has a complementary role in oxidative stress to combat the expected increase in ROS levels that occurs during fatty acid catabolism, preventing oxidation of cellular components. The human homologs of NHR-49, HNF-4α and PPARα, are well known regulators of energy metabolism, fatty acid uptake, lipoprotein transport, and mitochondrial and peroxisomal β-oxidation (Contreras *et al.* 2013). Knockdown of HNF4α in Caco-2 cells demonstrated increased lipid peroxidation and decreased antioxidant enzyme expression (Marcil *et al.* 2010), indicating that the role of NHR-49 in the oxidative stress response we describe is conserved between *C. elegans* and humans.

The genetic regulatory network uses positive and negative mechanisms to alter transcription, while at the same time coping with changes in the intracellular environment. We found that *nhr-49* was required for the increased expression of *skn-1c* observed in *brap-2* mutant animals, suggesting that *nhr-49* regulates influences SKN-1 levels and activity. It is possible that the SKN-1/MDT-15/NHR-49 complex is able to create a feed-forward mechanism that ensures SKN-1 is continuously produced in response to oxidative stress for the further downstream amplification of *gst-4* and phase II response genes (Figure 6). Our data provides insight into NHR-49 and its role in the oxidative stress response, where it influences stress response activation through co-regulation with SKN-1, an activation that requires a functional MDT-15. Although we were not able to detect direct binding between SKN-1 and NHR-49, we have shown that NHR-49 is required to coordinate with SKN-1 to induce the expression of stress genes.

In conclusion, we have established a new role for NHR-49 in the oxidative stress response in *C. elegans*. This result is in accord with recent studies published during the review/revision process (Qi *et al.* 2017; Goh *et al.* 2018) that *nhr-49* is required for the *skn-1* dependent oxidative stress response. Our work provides a framework for the continued study of stress genes and the ways in which they are regulated to maintain cell integrity and prevent damage caused by ROS.
